# Sudden death in epilepsy and ectopic neurohypophysis in Joubert syndrome 23 diagnosed using SNVs/indels and structural variants pipelines on WGS data: a case report

**DOI:** 10.1186/s12881-020-01024-y

**Published:** 2020-05-07

**Authors:** Dulika Sumathipala, Petter Strømme, Christian Gilissen, Ingunn Holm Einarsen, Hilde J. Bjørndalen, Andrés Server, Jordi Corominas, Bjørnar Hassel, Madeleine Fannemel, Doriana Misceo, Eirik Frengen

**Affiliations:** 1 Department of Medical Genetics, Oslo University Hospital and University of Oslo, Oslo, Norway; 2 Division of Pediatric and Adolescent Medicine, Oslo University Hospital, Oslo, Norway; 3 Faculty of Medicine, University of Oslo, Oslo, Norway; 4 Department of Human Genetics, Radboud UMC, Nijmegen, The Netherlands; 5 Section of Neuroradiology, Department of Radiology and Nuclear Medicine, Oslo University Hospital, Rikshospitalet, Oslo, Norway; 6 Department of Neurohabilitation and Complex Neurology, Oslo University Hospital, Ullevål, Oslo, Norway

**Keywords:** Case report, Ectopic neurohypophysis, Epilepsy, Joubert syndrome, *KIAA0586*, WGS

## Abstract

**Background:**

Joubert syndrome (JBTS) is a genetically heterogeneous group of neurodevelopmental syndromes caused by primary cilia dysfunction. Usually the neurological presentation starts with abnormal neonatal breathing followed by muscular hypotonia, psychomotor delay, and cerebellar ataxia. Cerebral MRI shows mid- and hindbrain anomalies including the molar tooth sign. We report a male patient with atypical presentation of Joubert syndrome type 23, thus expanding the phenotype.

**Case presentation:**

Clinical features were consistent with JBTS already from infancy, yet the syndrome was not suspected before cerebral MRI later in childhood showed the characteristic molar tooth sign and ectopic neurohypophysis. From age 11 years seizures developed and after few years became increasingly difficult to treat, also related to inadequate compliance to therapy. He died at 23 years of sudden unexpected death in epilepsy (SUDEP). The genetic diagnosis remained elusive for many years, despite extensive genetic testing. We reached the genetic diagnosis by performing whole genome sequencing of the family trio and analyzing the data with the combination of one analysis pipeline for single nucleotide variants (SNVs)/indels and one for structural variants (SVs). This lead to the identification of the most common variant detected in patients with JBTS23 (OMIM# 616490), rs534542684, in compound heterozygosity with a 8.3 kb deletion in *KIAA0586*, not previously reported.

**Conclusions:**

We describe for the first time ectopic neurohypophysis and SUDEP in JBTS23, expanding the phenotype of this condition and raising the attention on the possible severity of the epilepsy in this disease. We also highlight the diagnostic power of WGS, which efficiently detects SNVs/indels and in addition allows the identification of SVs.

## Background

Joubert syndrome (JBTS, OMIM# 213300) [1] is a rare disease with autosomal recessive inheritance, caused by the dysfunction of primary cilia and presenting as a neurodevelopmental syndrome [2]. Typical manifestations of the disease include neonatal breathing anomalies, hypotonia, cognitive impairment, cerebellar ataxia, and a complex mid- and hind-brain malformation visible as a molar tooth sign on axial cerebral MRI scans, which is a diagnostic signature finding in JBTS. Non-neurological manifestations, including cystic kidneys, liver disease, retinal dystrophy, chorioretinal colobomas, and polydactyly, may also be present. The disease is genetically heterogeneous with more than 35 genes currently known to cause it when mutated [3]. Among those genes, *KIAA0586* (OMIM# 610178) has been documented to cause JBTS23 (OMIM# 616490). Biallelic variants in *KIAA0586* are responsible of 2.5–7% of all patients with JBTS [4, 5]. In addition to JBTS23, dysfunction of KIAA0586 may cause a more severe ciliopathy known as Short-rib thoracic dysplasia 14 with polydactyly (SRTD14; OMIM# 616546), a complex syndrome with skeletal and neurological manifestations [6].

The KIAA0586 protein plays a central role in cilia formation. Primary cilia function as cellular antennae and are required for several signaling pathways essential for tissue growth and differentiation. KIAA0586 is a centrosomal protein, located at the distal ends of both the mother and daughter centrioless [7]. During cilia formation, KIAA0586 is crucial for the maturation of the mother centriole, through centriolar satellite dispersal and assembly of the basal body distal appendages, and basal body docking to the plasma membrane [8]. KIAA0586 functions upstream of the small GTPase Rab8 required for docking of the basal body to the plasma membrane [8, 9].

We present a Norwegian male patient with JBTS23. The genetic diagnosis was reached by performing whole genome sequencing (WGS) of the family trio, and analyzing the data with two distinct analysis pipelines one for single nucleotide variants (SNVs)/indels and one for structural variants (SVs). This allowed us to identify two pathogenic variants in *KIAA0586* and give the patient the genetic diagnosis of JBTS23. The patient developed epilepsy and died in early adulthood. Cerebral MRI revelead the presence of ectopic neurohypophisis, which has not been previously reported in JBTS23. We therefore expand the clinical phenotype and highlight the possible severity of epilepsy in JBTS23.

## Case presentation

**Fig. 1:**
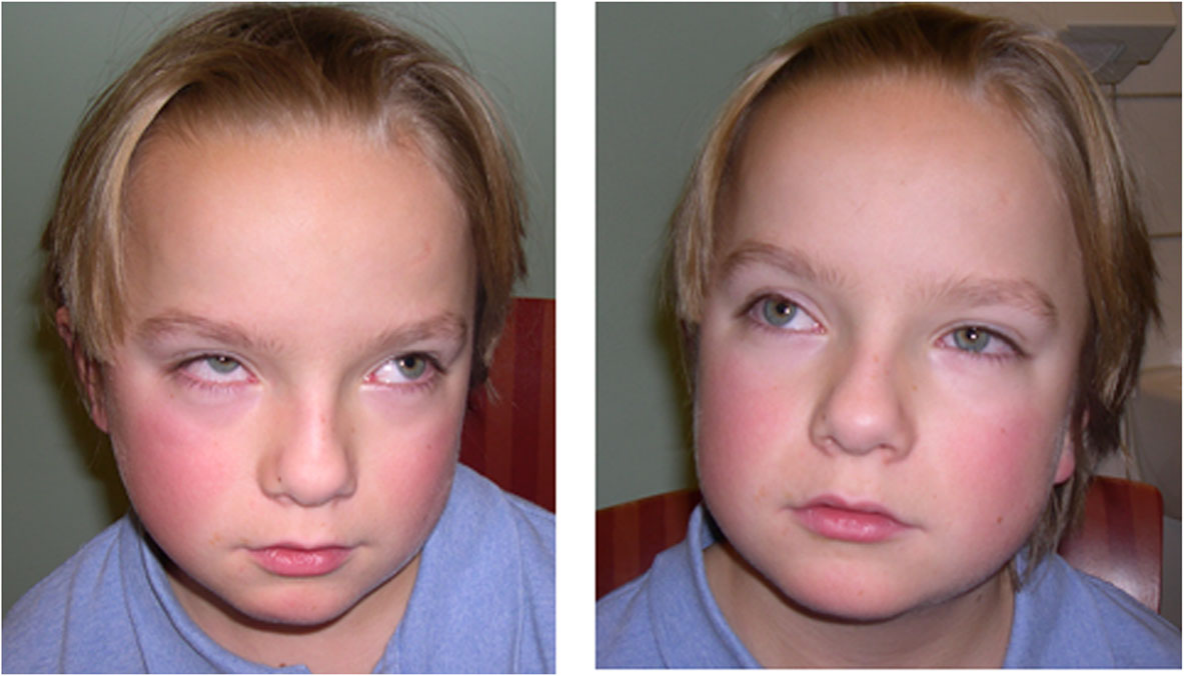
Photograph of the patient at the age of 11 years showing Duane (retraction) anomaly: when gazing to the left, the globe of the adducting right eye is retracting and when gazing to the right, the globe of the adducting left eye is retracting. Lateral eye movement to either side is limited because the corresponding abducens nerve nucleus inadequately innervates the lateral rectus muscle, resulting in globe retraction and narrowing of the palpebral fissure

**Fig. 2:**
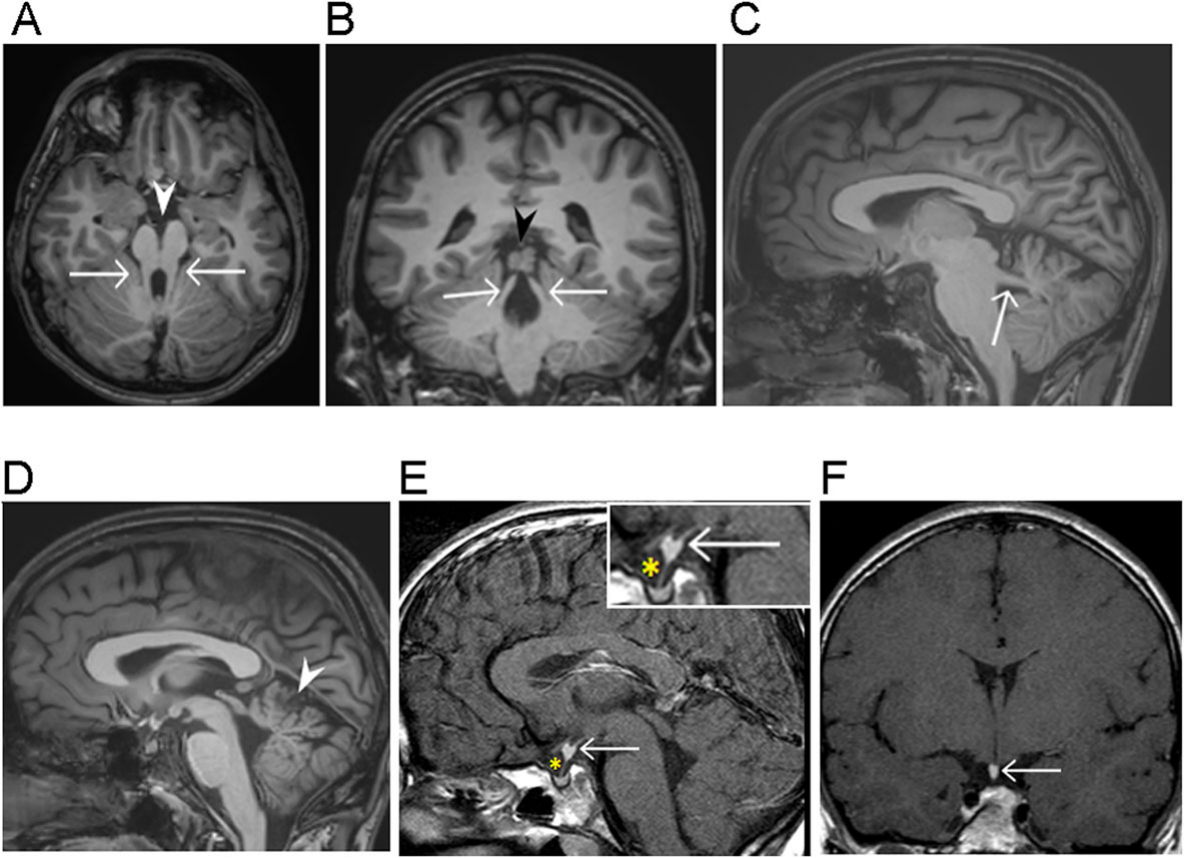
**a**-**d** Molar tooth sign in JBTS. Axial (**a**), coronal (**b**) and sagittal (**c**) T1-weighted images show large, thickened, elongated superior cerebellar peduncles (arrows). Note a deep interpeduncular fossa (white arrowhead) and the vermian cleft (black arrowhead). Midsagittal (**d**) T1-weighted image shows vermian hypoplasia (white arrowhead). **e-f** Ectopic posterior pituitary lobe. Postcontrast sagittal and coronal T1-weighted images show a posterior pituitary lobe (arrow) located at the level of the upper infundibulum. A very thin infundibulum is seen (asterisk) (magnified in the right upper corner of E).

We report a male patient who was the younger of two siblings born to non-consanguineous parents from Norway. The elder sibling and his parents were healthy. Initially, the patient presented with infantile episodes of apnea and tachypnea with hypotonia and global developmental delay. He manifested abnormal eye movements with oculomotor apraxia and Duane anomaly (Fig. 1), and cerebellar ataxia. Cerebral MRI showed the “molar tooth sign” characterized by elongated, thickened superior cerebellar peduncles, vermian hypoplasia and abnormal deep interpeduncular fossa (Fig.2a-d), neuroanatomical hallmarks of JBTS as well as ectopic neurohypophysis (Fig.2e-f). He had learning difficulties and when tested with a Wechsler Intelligence Scale for Children (WISC) he obtained a score of IQ 72. At 11 years he was diagnosed with epileptic seizures, at first only occurring at night, and had therefore escaped recognition for almost a year. He was admitted for the first time at 11 years and 9 months with a nocturnal generalized tonic clonic seizure (GTCS) and was started on Oxcarbazepine, which kept him almost seizure free for several years. This drug was tapered at 18 y, but soon after his epilepsy recurred, now manifesting both as GTCS and absences. Oxcarbazepine was reinstituted, but absences, sometimes with myoclonus, persisted, and treatment was changed to Valproate. However, GTCS increased in frequency, often followed by prolonged headaches that did not clear before the next day. His treatment was then changed to Lamotrigine, which he however was reluctant to take. He was living alone and managed to attend shelter work. At 22 years he was recognized to have two seizure types, GTCS and sensory epilepsy, the latter manifesting as abrupt abdominal discomfort, considered to be of temporal lobe origin. Nocturnal GTCS occurred 2–3 times per week and were often initiated by yawning and drooling and ending with vomiting, followed by drowsiness for the rest of the day. At 23 years one morning he was found dead in bed and according to his mother he laid in a flexed position that was usual for him after having a seizure. Postmortem examination concluded with no trace of Lamotrigine in his blood. The fatal outcome was classified as sudden unexpected death in epilepsy (SUDEP) [10], and was suspected to have been precipitated by the lack of compliance to anticonvulsive treatment.

## Genetic investigations

**Fig. 3:**
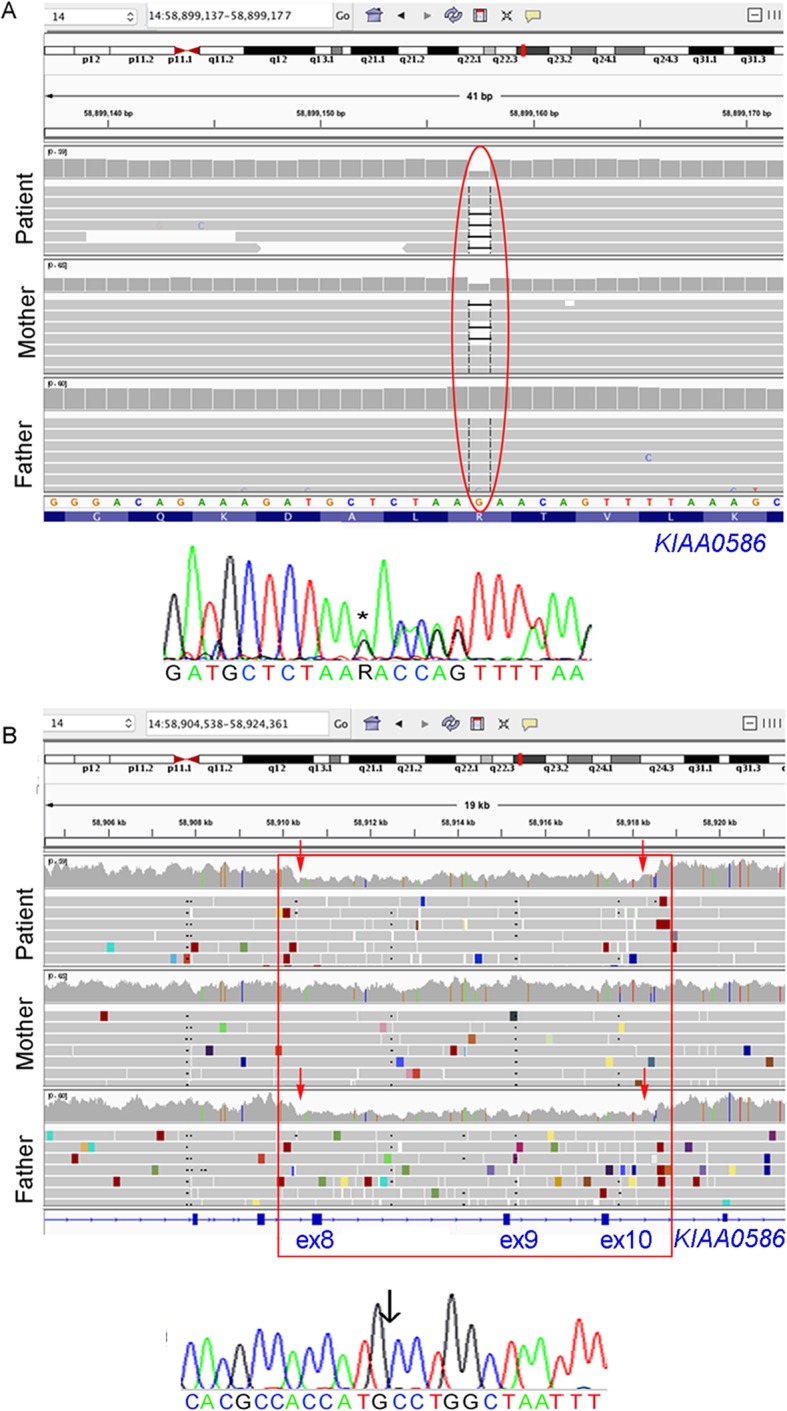
**a** Upper. WGS data in Integrative Genome Viewer (IGV) (http://software.broadinstitute.org/software/igv) showing the heterozygous 1 bp deletion in *KIAA0586* in the patient and his mother, but not in the father (red oval). Lower. Sanger sequencing on DNA from blood of the patient verifying 1 bp deletion in *KIAA0586.* R = A or G (as a consequence of the heterozygous deletion of G followed by A). **b** Upper. Screenshot from the IGV showing the 8.3 kb deletion in *KIAA0586* detected in heterozygosity in the patient and his father, but not in the mother (red square) removing exons 8, 9 and 10 in *KIAA0586* (blue boxes in the bottom). Note the decreased coverage in the deleted region in the patient and his father (red vertical arrows) and the reads with ends spanning the deletions (in red). Lower. Sanger sequencing of DNA from blood of the patient verified the 8.3 kb deletion in *KIAA0586* and re-defined its breakpoints slightly to chr14 (GRCh37):g.58910301–58,918,610.

Microarray and whole exome sequencing (WES) data analysis of the family trio did not identify any putative pathogenic variants explaining the disease (data not shown); therefore, we performed WGS of the family trio (methods are described in the Additional file 1). We reached the genetic diagnosis of the patient combining two WGS data analysis pipelines. First, we analyzed SNVs/indels, which were called on the patient-parent trio data using Genome Analysis Toolkit (GATK) v.3.4. Haplotype Caller in GVCF model. The first analysis with this pipeline identified a maternally inherited frameshift variant in *KIAA0586* (NM_001244189), chr14 (GRCh37):g.58899157delG (c.428delG) (Fig. 3a), rs534542684, predicted to form a premature stop codon (p.Arg143Lysfs*4), which was verified by Sanger sequencing. This variant, was previously reported as pathogenic [5, 11–13]. Pathogenic variants in *KIAA0586* cause two distinct autosomal recessive diseases [4–7, 11–13]. One is the Short-rib thoracic dysplasia 14 with polydactyly (SRTD14; OMIM# 616546), a complex syndrome with skeletal and neurological manifestations [6]. The other is JBTS23 (OMIM# 616490). As the variant could not explain the recessive phenotype, we subsequently used a data analysis pipeline for structural variants (details in the Supplementary information), which detected a paternally inherited 8.3 kb deletion in chr14 (GRCh37):g.58910278–58918611 according to variant calling of the high throughput sequencing data. The deletion lead to removal of exons 8, 9 and 10 in *KIAA0586* (NM_001244189), predicted to cause direct splicing of exon 7 to 11 in the transcript, leading to a frameshift and formation of a premature stop codon (p.Val249Glufs*3). Deletions of this genomic region has not previously been described (Database of Genomic Variants, http://dgv.tcag.ca and Decipher, https://decpher.sanger.ac.uk). Sanger sequencing analysis verified the segregation of this deletion with the disease in the family and re-defined the breakpoints of the SV slightly to chr14 (GRCh37):g.58910301–58918610 (Fig. 3b). The discrepancy in the break-end calculation can be attributed to the Manta base-pair resolution capability [14]. The clinical presentation of the patient was compatible with JBTS23. Thus, we identified pathogenic compound heterozygous variants in *KIAA0586* by applying two distinct analysis pipelines on the WGS data, and confirmed the patient with JBTS23. We did not identify additional deleterious variants in other known JBTS causing genes in WES or WGS data.

## Discussion and conclusions

We report a patient with biallelic variants in *KIAA0586*, causing JBTS23. The maternal *KIAA0586* variant, c.428delG, known as rs534542684, is reported with minor allele frequency (MAF) 0.003 in the Exome Aggregate Consortium (ExAC) and in the Genome Aggregation database (gnomAD). Surprisingly, this variant has been identified in homozygosity in two healthy individuals in the European population in gnomAD and in one healthy homozygous female. In this female the *KIAA0586* c.428delG transcript was shown to elude the nonsense-mediated decay (NMD) mechanism [12]. It was suggested that the allele carrying rs534542684 could function as a hypomorphic allele, possibly by the use of an alternative start codon downstream of the variant [12]. It is interesting to note that c.428delG is the most frequently identified variant in JBTS23, usually present in compound heterozygosity, less often in homozygosity [5, 13]. In our patient the second allele harbored a deletion removing exons 8–10.

Patients with JBTS23 present with neonatal breathing pattern anomalies, global developmental delay, intellectual disability, and brain malformations, including the molar tooth sign. In addition to the typical JBTS23 presentation, our patient manifested with Duane anomaly, previously reported only in one case [13]. Interestingly, the patient suffered from epilepsy and had an ectopic neurohypophysis. The last feature has never been reported in patients with JBTS23.

Epilepsy is reported only in patients with mutations in five of the known JBTS causing genes: *CC2D2A* causing JBTS9 (OMIM# 612285), *KIF7* causing JBTS12 (OMIM# 200990), *KIAA0586* causing JBTS23 (OMIM# 616490), *ARMC9* causing JBTS30 (OMIM# 617622), and *B9D2* causing (OMIM# 614175). None of the animal models targeting *KIAA0586* orthologues so far reported manifested epilepsy [15]. Among the 42 JBTS23 patients so far reported [4, 5, 7, 11–13], only two suffered from epilepsy [7, 11], and SUDEP has not been reported. However, the patient presented several clinical risk factors for the occurrence of SUDEP, such as being a male with a history of seizures from a young age, often nocturnal GTCS, borderline intellectual disability, and living alone not complying to the treatment [10]. In particular, the combination of frequent nocturnal GTCS and sleeping alone was showed to dramatically increase the risk of SUDEP [16]. In general mortality in JBTS has not been related to epilepsy, but rather to renal or respiratory failure [17], however the recent management recommendations for patients with JBTS specifically mention seizures [3].

Our patient is the first to be reported with deleterious variants in *KIAA0586* and ectopic neurohypophysis. The ectopic neurohypophysis is a midline brain malformation consisting of an aberrant pituitary development with an ectopically located posterior pituitary gland. This brain malformation can be associated with endocrinological defects ranging from isolated growth hormone deficiency to multiple anterior pituitary hormone deficiencies, but posterior pituitary function remains unchanged. However, our patient had a normal endocrinological profile. It was proposed that the occurrence of the ectopic neurohypophysis in ciliopathies might be caused by the role of the cilia in influencing pituitary development through SHH and Wnt signaling pathways [18].

In conclusion, we identified compound heterozygous variants in *KIAA0586* in a patient with JBTS23, presenting with ectopic neurohypophysis and juvenile onset of epilepsy, which was difficult to treat and resulted in SUDEP. We expand the phenotype of JBTS23 since these features have not previously been reported in this disease. Although we report a single patient, we suggest that the onset of epilepsy in patients with JBTS23 should be promptly evaluated and treated, and deserve close monitoring, to reduce the risk of an adverse outcome. Our results highlight the value of WGS when combining different pipelines of analysis to detect SNVs/indels and larger structural variants below the resolution of diagnostic microarrays.

## Supplementary information

**Additional file 1.** Details of the methods for the genetic investigations.

## Data Availability

Data available on request, but may be subjected to restrictions due to privacy/ethical restrictions.

## Abbreviations

IGV: Integrative genome viewer
JBTS: Joubert syndrome
OMIM: Online Mendelian inheritance in men
SNVs: Single nucleotide variants
SUDEP: Sudden unexpected death in epilepsy
SVs: Structural variants
WES: Whole exome sequencing
WGS: Whole genome sequencing
