# Supplementary material for: Sudden death in epilepsy and ectopic neurohypophysis in Joubert syndrome 23 diagnosed using SNVs/indels and structural variants pipelines on WGS data: a case report
Source: BMC Med Genet. 2020 May 7;21:96. doi: 10.1186/s12881-020-01024-y (PMC7204034; doi:10.1186/s12881-020-01024-y)
Supplement: Supplementary file 1 — Additional file 1. Details of the methods for the genetic investigations. [file 12881_2020_1024_MOESM1_ESM.docx]

**Supplemental data**

**Sudden death in epilepsy and ectopic neurohypophysis in Joubert syndrome 23 diagnosed using SNVs/indels and structural variants pipelines on WGS data**

**Supplemental methods**

**Whole genome sequencing (WGS) and analysis of the SNVs/Indels**

Genomic DNA was extracted from peripheral blood in the parents and patient. Sample preparation was done using TruSeq^TM^ PCR-free prep. Whole genome sequencing (WGS) of the patient and his parents was performed using the Illumina HiSeqX instrument (Illumina, San Diego, CA) with 350 bp paired-end reads. Alignment against the GRCh37 human reference genome was performed with a Burrows-Wheeler Aligner (BWA) v.0.7.8. (1) PCR-duplicates marking and removal with Picard v.1.119 (http://broadinstitute.github.io/picard/), indel realignment, base quality recalibration, and joint variant calling was done with the Genome Analysis Toolkit (GATK) v.3.4 (2, 3). Functional annotation was performed with Variant Effect Predictor (VEP) tool in Ensembl 85. (4) Variants with a high probability of being technical artifacts, as computed by the GATKs "variant quality score recalibration" procedure, were removed in the filtering process. The three variant calling files (VCFs) generated were simultaneously analyzed using the FILTUS program (5). We discarded variants with allelic frequency >0.01 in any of the databases used (GnomAD, gnomad.broadinstitute.org/; ExAC, exac.broadinstitute.org/; 1000Genomes, internationalgenome.org). We also discarded variants *in silico* predicted as benign/tolerated for protein function according to the Combined Annotation Dependent Depletion (CADD) (CADD phred score <15) (6). We focused on missense, nonsense, frameshift, and small insertion/deletion variants. Genome data were analyzed with autosomal recessive (homozygous and compound heterozygous) and autosomal dominant mode of inheritance.

**Structural variant pipeline**

Structural variants (SVs) where called from mapped paired-end sequencing reads by using Manta 1.1.0 (7). Manta uses both paired read fragment spanning and split read evidence to call translocations, deletions, tandem duplications, insertions and inversions. Standard Manta quality checks were applied and variants that pass all default filters were selected. Raw data files were annotated using an in-house pipeline. Potential structural variants were analysed against selected used public databases: Decipher (decipher.sanger.ac.uk), Wellderly (genomics.scripps.edu/browser), GoNL (nlgenome.nl), 1000G (internationalgenome.org), and DGV (dgv.tcag.ca/dgv/app/home).

**PCR and Sanger sequencing**

Genomic DNA from peripheral blood of the family was used to generate PCR products with primers flanking the variants identified in *KIAA0586* (NM_001244189).

PCR products were purified and Sanger sequenced using an ABI 3730xl DNA analyzer and ABI BigDye dye terminator cycle-sequencing kits v3.1 (Life Technologies, Carlsbad, CA). Sequences were analyzed with DNA Sequencing Analysis Software v. 5.1 (Applied Biosystems, Foster City, CA) and SeqScape Software v.2.7 (Thermo Fisher Scientific).

The following primers were used: 1) Fwd 5’ TGGCAGATTTGTTTGAGACG -3’ and Rev 5’- CACAAGTCCCCAAAGTCCAT -3` (to verify the 8.3 kb deletion);

2) Fwd 5’- TTGCAGCAAATGACATCTTCA -3’ and Rev 5’- ACGTGTCTAGCACCATGAGAAA -3’ (to verify the 1 bp deletion). The primers map to the following region chr14: 58899100-58899120 and chr14:58899299-58899320 in USCS browser GRCh37.

**References**

1. Li H, Durbin R. Fast and accurate short read alignment with Burrows-Wheeler transform. Bioinformatics. 2009;25(14):1754-60.

2. McKenna A, Hanna M, Banks E, Sivachenko A, Cibulskis K, Kernytsky A, et al. The Genome Analysis Toolkit: a MapReduce framework for analyzing next-generation DNA sequencing data. Genome Res. 2010;20(9):1297-303.

3. DePristo MA, Banks E, Poplin R, Garimella KV, Maguire JR, Hartl C, et al. A framework for variation discovery and genotyping using next-generation DNA sequencing data. Nat Genet. 2011;43(5):491-8.

4. McLaren W, Gil L, Hunt SE, Riat HS, Ritchie GR, Thormann A, et al. The Ensembl Variant Effect Predictor. Genome Biol. 2016;17(1):122.

5. Vigeland MD, Gjotterud KS, Selmer KK. FILTUS: a desktop GUI for fast and efficient detection of disease-causing variants, including a novel autozygosity detector. Bioinformatics. 2016;32(10):1592-4.

6. Kircher M, Witten DM, Jain P, O'Roak BJ, Cooper GM, Shendure J. A general framework for estimating the relative pathogenicity of human genetic variants. Nat Genet. 2014;46(3):310-5.

7. Chen X, Schulz-Trieglaff O, Shaw R, Barnes B, Schlesinger F, Kallberg M, et al. Manta: rapid detection of structural variants and indels for germline and cancer sequencing applications. Bioinformatics. 2016;32(8):1220-2.
